# Clinical effects of persistent pulmonary hypertension in newborns diagnosed with transposition of the great arteries

**DOI:** 10.3389/fcvm.2026.1888066

**Published:** 2026-07-23

**Authors:** Halise Zeynep Genc, Huseyin Ceylan, Hacer Kamali, Eymen Recep, Ali Can Hatemi, Sertac Haydin, Alper Guzeltas, Erkut Ozturk

**Affiliations:** 1 Department of Pediatric Cardiology, Saglik Bilimleri University Basaksehir Cam and Sakura City Hospital, Istanbul, Turkey; 2 Department of Pediatric Cardiology, Saglik Bilimleri University Mehmet Akif Ersoy Thoracic and Cardiovascular Surgery Education and Research Hospital, Istanbul, Turkey; 3 Department of Pediatric Cardiovascular Surgery, Saglik Bilimleri University Basaksehir Cam and Sakura City Hospital, Istanbul, Turkey; 4 Department of Pediatric Cardiovascular Surgery, Saglik Bilimleri University Mehmet Akif Ersoy Thoracic and Cardiovascular Surgery Education and Research Hospital, Istanbul, Turkey

**Keywords:** arterial switch operation (ASO), neonate, perioperative outcomes, persistent pulmonary hypertension of the newborn (PPHN), transposition of the great arteries (TGA)

## Abstract

**Introduction:**

Transposition of the great arteries (TGA) is one of the critical congenital heart diseases of the newborn. The transition from fetal to postnatal circulation in these infants is complex, and the presence of persistent pulmonary hypertension of the newborn (PPHN) during this period may affect surgical outcomes. This study aims to evaluate the impact of PPHN on perioperative morbidity and mortality in infants with TGA.

**Materials and methods:**

The study was conducted between December 1, 2024, and December 1, 2025, in two high-volume cardiac centers, involving newborns diagnosed with TGA. Patients were divided into two groups—PPHN and non-PPHN—based on clinical and echocardiographic findings. The groups were evaluated for early-term (first 30 days) mortality and morbidity. Findings were analyzed statistically.

**Results:**

A total of 48 newborns with TGA (60% male) were included. The median age at operation was 5 days (IQR 3–7), median weight was 3,100 g (IQR 2,900–3,400), and the median preoperative oxygen saturation was 76% (IQR 70–82). Balloon atrial septostomy was performed in eight infants prior to ASO. PPHN was identified in thirteen infants (27%). Postoperative complication rates—including duration of vasopressor use, duration of sedative medication, length of hospital stay, and duration of mechanical ventilation—were higher in the PPHN group (*p* < 0.05). Overall mortality was 10.4%, with no significant difference between the PPHN and non-PPHN groups (15.3% vs. 8.5%; *p* = 0.60).

**Conclusion:**

In newborns with TGA, PPHN was associated with increased postoperative morbidity. While no statistically significant difference in early mortality was observed, larger multicenter studies are needed to better define the impact of PPHN on mortality risk.

## Introduction

Transposition of the great arteries (TGA) is one of the most critical congenital heart diseases presenting in the neonatal period and is characterized by ventriculoarterial discordance resulting in parallel systemic and pulmonary circulations (1). Since the introduction of the arterial switch operation (ASO) by Adib Jatene, early survival has improved dramatically, with contemporary series reporting survival rates exceeding 90% (2).

Persistent pulmonary hypertension of the newborn (PPHN) represents a failure of normal postnatal circulatory transition and is characterized by elevated pulmonary vascular resistance leading to right-to-left shunting and severe hypoxemia (3). In neonates with TGA, the coexistence of PPHN may exacerbate hypoxemia, impair left ventricular preparedness, and complicate perioperative management. Earlier studies reported that severe pulmonary hypertension in TGA was associated with increased preoperative instability and mortality despite inhaled nitric oxide therapy (4, 5).

However, more recent data suggest that with modern intensive care strategies—including prostaglandin E1 infusion, balloon atrial septostomy, optimized ventilation, and pulmonary vasodilator therapy—the impact of PPHN on perioperative mortality may be attenuated (6).

Given these conflicting findings, we aimed to evaluate the impact of PPHN on perioperative morbidity and early mortality in neonates with TGA undergoing ASO.

## Methods

This retrospective multicenter cohort study was conducted in two high-volume tertiary congenital cardiac centers between December 1, 2024, and December 1, 2025. The study protocol was approved by the Ethics Committee of Basaksehir Cam and Sakura City Hospital, and the approved protocol specifically covered data collection from both participating centers: Basaksehir Cam and Sakura City Hospital and Mehmet Akif Ersoy Thoracic and Cardiovascular Surgery Training and Research Hospital. The study was conducted in accordance with the principles of the Declaration of Helsinki. All consecutive term and near-term neonates diagnosed with dextro-transposition of the great arteries (d-TGA) who underwent arterial switch operation (ASO) were eligible. Diagnosis was confirmed by transthoracic echocardiography. Exclusion criteria included:

- TGA with major associated cardiac anomalies requiring alternative surgical strategies
- Major chromosomal abnormalities
- Incomplete clinical records
- Taussig–Bing physiology

### Definition of PPHN

PPHN was diagnosed using both clinical and echocardiographic criteria. Clinically, infants were required to demonstrate a preductal (right arm)—postductal (lower extremity) transcutaneous oxygen saturation difference greater than 5% while receiving 100% oxygen. In neonates with d-TGA and suspected PPHN, reverse differential cyanosis can be observed. Consequently, preductal oxygen saturation measured in the right upper extremity may be lower than postductal saturation measured in the lower extremities, reflecting the unique parallel circulation physiology and ductal shunting characteristics of this lesion. Echocardiographic confirmation included at least one of the following findings: right-to-left or bidirectional shunting across the patent ductus arteriosus and/or foramen ovale, flattening or leftward bowing of the interventricular septum consistent with right ventricular pressure overload, tricuspid regurgitation velocity suggestive of elevated pulmonary artery pressure (typically >3 m/s when measurable), or an estimated pulmonary artery pressure ≥ two-thirds of systemic arterial pressure, particularly when approaching or exceeding systemic pressure. Final classification of PPHN was based on the combined clinical and echocardiographic assessment (6). Diagnosis was based on oxygenation criteria consistent with established neonatal PPHN definitions.

### Perioperative management

All patients received prostaglandin E1 infusion following diagnosis to maintain ductal patency and optimize systemic oxygen delivery. Balloon atrial septostomy was performed only in patients with restrictive interatrial communication and inadequate mixing resulting in persistent hypoxemia despite prostaglandin E1 therapy. Inhaled nitric oxide was initiated in neonates with clinical and echocardiographic evidence of pulmonary hypertension associated with significant hypoxemia despite optimized ventilatory and hemodynamic support.

Pulmonary hypertensive crisis was defined as an acute episode of severe hypoxemia and/or hemodynamic instability associated with suspected abrupt elevation of pulmonary vascular resistance requiring escalation of pulmonary vasodilator therapy and intensive cardiovascular support. During iNO therapy, nitric oxide was introduced into the inspiratory limb of the ventilator (10–20 ppm). In patients receiving inhaled nitric oxide therapy, oral sildenafil was initiated at a dose of 2 mg/kg/day approximately 24 h before planned discontinuation of nitric oxide in order to facilitate weaning and reduce the risk of rebound pulmonary hypertension.

Extracorporeal membrane oxygenation (ECMO) was considered in cases of refractory hypoxemia, severe ventricular dysfunction, or persistent hemodynamic instability despite maximal medical management.

In summary, pulmonary vasodilator and mechanical circulatory support therapies were applied according to predefined institutional protocols. Inhaled nitric oxide was used as first-line pulmonary vasodilator therapy, sildenafil was introduced to facilitate nitric oxide weaning and reduce the risk of rebound pulmonary hypertension, and ECMO was reserved for refractory hypoxemia, severe ventricular dysfunction, or persistent hemodynamic instability despite maximal medical therapy.

### Variables collected

Demographic and clinical variables included: gestational age, birth weight, mode of delivery, preoperative prostaglandin E1 use, balloon atrial septostomy, inhaled nitric oxide (iNO) therapy, ECMO requirement, duration of mechanical ventilation, duration of vasoactive support, ICU length of stay, total hospital stay, and early mortality (≤30 days).

To minimize selection bias, all consecutive eligible neonates undergoing arterial switch operation during the study period at both participating centers were included in the analysis.

### Statistical analysis

Continuous variables were tested for normality using Shapiro–Wilk test. Non-normally distributed variables were expressed as median (IQR) and compared using Mann–Whitney *U* test. Categorical variables were compared using chi-square or Fisher's exact test. Statistical significance was defined as *p* < 0.05.

## Results

During the study period, 52 neonates with d-transposition of the great arteries (d-TGA) were screened. Four patients were excluded due to incomplete data, leaving 48 neonates for final analysis. Of these, 13 (27%) were diagnosed with persistent pulmonary hypertension of the newborn (PPHN) and 35 (73%) comprised the non-PPHN group.

Baseline characteristics are summarized in Table 1. The overall cohort was 60% male, with a median gestational age of 38 weeks (IQR 37–39) and a median birth weight of 3,100 g (IQR 2,900–3,400). Demographic variables, including sex distribution, gestational age, birth weight, mode of delivery, and prevalence of low birth weight (<2,500 g), were comparable between groups (all *p* > 0.05).

**Table 1:** Baseline characteristics of the study population.

Variable	Total (*n* = 48)	PPHN (*n* = 13)	Non-PPHN (*n* = 35)	*p* value
Male sex	29 (60%)	8 (61.5%)	21 (60%)	0.92
Gestational age (weeks)	38 (37–39)	38 (37–39)	38 (37–39)	0.88
Birth weight (g)	3,100 (2,900–3,400)	3,000 (2,850–3,300)	3,150 (2,950–3,450)	0.27
Low birth weight (<2,500 g)	5 (10.4%)	2 (15.3%)	3 (8.5%)	0.61
Cesarean delivery	31 (64.5%)	9 (69.2%)	22 (62.8%)	0.69
Preoperative SpO_2_ (%)	76 (70–82)	72 (68–78)	78 (72–84)	**0.03**
Prostaglandin E1 use	48 (100%)	13 (100%)	35 (100%)	–
Balloon atrial septostomy	8 (16.6%)	3 (23.0%)	5 (14.2%)	0.72
Inhaled nitric oxide (preop)	11 (22.9%)	8 (61.5%)	3 (8.5%)	**0.004**
ECMO (preop)	2 (4.1%)	1 (7.6%)	1 (2.8%)	0.48
Age at ASO (days)	5 (3–7)	5 (3–8)	5 (3–7)	0.81
Ventricular septal defect	8 (16.6%)	2 (15.3%)	6 (17.1%)	0.92
Abnormal coronary anatomy	17 (35%)	5 (38%)	12 (34%)	0.65

Data presented as median (IQR) or *n* (%). SpO_2_, transcutaneous oxygen saturation; ASO, arterial switch operation; ECMO, extracorporeal membrane oxygenation.

Bold values indicate statistically significant differences (*p* < 0.05).

Median preoperative oxygen saturation was significantly lower in the PPHN group (72% [IQR 68–78]) compared with the non-PPHN group (78% [IQR 72–84]; *p* = 0.03). All patients received prostaglandin E1 infusion preoperatively. Balloon atrial septostomy was performed in eight patients (16.6%) without intergroup difference (*p* = 0.72).

Preoperative inhaled nitric oxide therapy was significantly more frequent in the PPHN group (61.5% vs. 8.5%; *p* = 0.004). Preoperative ECMO support was rare and did not differ significantly between groups. The median age at arterial switch operation was 5 days (IQR 3–7), with no significant difference between groups (*p* = 0.81).

Postoperative outcomes are summarized in Table 2. Neonates with PPHN required significantly greater postoperative support compared with those without PPHN. The duration of mechanical ventilation was significantly longer in the PPHN group (7 [5–11] vs. 4 [3–6] days; *p* = 0.002). Similarly, the duration of vasoactive support was prolonged (6 [4–9] vs. 3 [2–5] days; *p* = 0.001). Intensive care unit length of stay was also significantly extended in infants with PPHN (12 [9–18] vs. 7 [5–10] days; *p* = 0.001), as was total hospital stay (20 [16–28] vs. 14 [11–18] days; *p* = 0.003). Pulmonary hypertensive crises occurred in three patients, all of whom were in the PPHN group (23.0% vs. 0%; *p* = 0.01). Among the three patients who developed a pulmonary hypertensive crisis, two had previously undergone balloon atrial septostomy, and one underwent arterial switch operation 1 day after the crisis following clinical stabilization. Although postoperative ECMO support was required in three patients overall (6.2%), the difference between groups did not reach statistical significance (15.3% vs. 2.8%; *p* = 0.18).

**Table 2:** Operative and postoperative outcomes.

Outcome	PPHN (*n* = 13)	Non-PPHN (*n* = 35)	*p* value
CPB duration (minutes)	110 (90–130)	120 (100–135)	0.214
Mechanical ventilation (days)	7 (5–11)	4 (3–6)	**0.002**
Vasoactive support (days)	6 (4–9)	3 (2–5)	**0.001**
VIS at 24 h, median (IQR)	9 (7–12)	7 (5–10)	0.06
Delayed sternal closure	2 (15.3%)	6 (17.1%)	0.87
ICU length of stay (days)	12 (9–18)	7 (5–10)	**0.001**
Total hospital stay (days)	20 (16–28)	14 (11–18)	**0.003**
Pulmonary hypertensive crisis	3 (23.0%)	0	**0.01**
Postoperative ECMO	2 (15.3%)	1 (2.8%)	0.18
Early mortality (≤30 days)	2 (15.3%)	3 (8.5%)	0.60

Data presented as median (IQR) or *n* (%). CPB, cardiopulmonary bypass; ICU, intensive care unit; VIS, vasoactive-inotropic score.

Bold values indicate statistically significant differences (*p* < 0.05).

Preoperative ECMO support was required in two patients because of refractory hypoxemia accompanied by severe hemodynamic compromise. Postoperative ECMO was initiated in three patients, primarily because of severe ventricular dysfunction and persistent hemodynamic instability despite maximal pharmacologic support.

Overall early mortality (≤30 days) was 10.4% (5/48). Mortality was numerically higher in the PPHN group (15.3%) compared with the non-PPHN group (8.5%); however, this difference was not statistically significant (*p* = 0.60).

Because only five mortality events occurred during the study period, reliable identification of independent predictors of mortality was not feasible. Therefore, no multivariable mortality analysis was performed.

## Discussion

In this multicenter cohort, PPHN was primarily associated with increased postoperative morbidity, including prolonged mechanical ventilation, longer vasoactive support, and extended ICU and hospital stay, rather than a measurable increase in early mortality. Improved contemporary management of persistent pulmonary hypertension, including pulmonary vasodilator therapy, optimized ventilatory support, and timely surgical intervention, may have contributed to the absence of a significant difference in early postoperative mortality among neonates with d-transposition of the great arteries. However, because of the limited number of mortality events, the present study may have been underpowered to detect a clinically meaningful effect of PPHN on mortality.

The pathophysiology of PPHN involves failure of normal pulmonary vascular transition after birth, resulting in persistently elevated pulmonary vascular resistance (PVR) and right-to-left shunting, as previously described (3, 7). In neonates with d-TGA, where systemic and pulmonary circulations function in parallel, elevated PVR may further compromise effective oxygen delivery and exacerbate perioperative instability.

An important consideration is whether PPHN itself directly contributes to adverse postoperative outcomes or primarily serves as a marker of greater physiological compromise before surgery. In our cohort, patients with PPHN demonstrated lower preoperative oxygen saturation and substantially higher use of inhaled nitric oxide, suggesting more severe cardiopulmonary instability. Therefore, the observed increase in postoperative morbidity may reflect both the pathophysiological effects of pulmonary hypertension and the overall severity of the preoperative condition. Consequently, PPHN may serve not only as a potential contributor to postoperative morbidity but also as a marker of greater preoperative physiological compromise, and the observed associations should not be interpreted as evidence of a direct causal relationship.

In contrast to Modestini et al. (6), our findings show that persistent pulmonary hypertension of the newborn significantly increases postoperative morbidity in TGA. Patients with PPHN required longer mechanical ventilation and vasoactive support and had prolonged ICU and hospital stays. In contrast, cardiopulmonary bypass duration was similar between groups and was numerically longer in the non-PPHN group, suggesting that operative complexity alone is unlikely to explain the increased postoperative morbidity observed in the PPHN cohort. Although the 24-hour vasoactive-inotropic score (VIS) did not reach statistical significance, a trend toward higher VIS values was observed in the PPHN group, consistent with greater postoperative cardiovascular support requirements. However, consistent with Modestini et al., early mortality was similar between groups. These results suggest that while we did not detect a significant effect of PPHN on early survival, PPHN remains associated with increased postoperative morbidity and resource utilization. While Modestini et al. primarily focused on the impact of PPHN on mortality and major perioperative outcomes, the present study extends these observations by providing a more detailed characterization of postoperative morbidity. Specifically, we evaluated duration of mechanical ventilation, vasoactive support, ICU stay, hospital stay, and pulmonary hypertensive crises, thereby offering a broader assessment of the perioperative burden associated with PPHN. In addition, our findings represent a contemporary experience from two high-volume congenital cardiac centers and contribute further evidence regarding the clinical impact of PPHN in neonates undergoing arterial switch operation.

Experimental and translational evidence from Abman (8) and Lakshminrusimha (9) has shown that hypoxemia, acidosis, and inflammatory activation enhance pulmonary vasoreactivity, predisposing susceptible neonates to pulmonary hypertensive crises. In our cohort, pulmonary hypertensive crises occurred exclusively in the PPHN group, further supporting this pathophysiological framework.

Although crude mortality was numerically higher in the PPHN group, no statistically significant difference in early mortality was detected between groups. Given the limited number of mortality events, the study may have been underpowered to detect clinically meaningful differences in mortality risk. Contemporary arterial switch operation (ASO) series have reported excellent survival outcomes. Fricke et al. (4), Khairy et al. (2), and Santens et al. (10) demonstrated survival rates exceeding 90% in modern cohorts. These improvements likely reflect advances in early diagnosis, perioperative management, surgical technique, and postoperative critical care.

Earlier literature suggested a stronger association between pulmonary hypertension and mortality in TGA. Sallaam et al. (11) reported increased perioperative mortality in neonates with significant pulmonary hypertension. However, differences in era, patient selection, and perioperative protocols may explain the discrepancy with contemporary findings, including ours and those of Modestini et al. (6).

Advances in neonatal management have likely attenuated the mortality impact of PPHN. Balloon atrial septostomy, first described by Rashkind and Miller (12), remains essential for improving intercirculatory mixing in d-TGA. Selective pulmonary vasodilation with inhaled nitric oxide, as outlined by Kinsella (13), has significantly improved oxygenation in neonates with pulmonary hypertension.

Risk stratification tools such as the vasoactive-inotropic score (VIS), described by Gaies et al. (14), have enhanced early identification of patients at risk for prolonged ICU courses. These integrated strategies likely explain why PPHN today manifests primarily as increased morbidity rather than mortality.

Our findings, in concordance with those of Modestini et al. (6), suggest that PPHN in neonates with d-TGA is associated with increased perioperative complexity and postoperative morbidity. However, the current study was not sufficiently powered to definitively determine the impact of PPHN on mortality. Larger multicenter investigations are required to clarify whether PPHN independently contributes to mortality risk in this population. Early recognition, aggressive pulmonary vasodilator therapy, and optimized hemodynamic support remain essential to minimize ICU burden.

## Limitations

This study is limited by its retrospective design and moderate sample size. Direct invasive measurements of pulmonary artery pressure were not systematically available. Diagnostic criteria relied on clinical and echocardiographic parameters consistent with established definitions. In addition, only five mortality events occurred during the study period. Consequently, reliable identification of independent predictors of mortality was not feasible, and the study may have been underpowered to detect clinically meaningful differences in mortality between groups. Similarly, because only three patients experienced a pulmonary hypertensive crisis, a detailed subgroup analysis of these cases was not feasible. Finally, multiple comparisons were performed, and therefore some statistically significant findings may have occurred by chance.

## Conclusion

Persistent pulmonary hypertension of the newborn was associated with increased postoperative morbidity in neonates undergoing arterial switch operation for d-transposition of the great arteries. Although no statistically significant association with early mortality was detected, the limited sample size and low number of mortality events preclude definitive conclusions regarding mortality risk. Furthermore, PPHN may represent not only a potential contributor to postoperative morbidity but also a marker of greater preoperative physiological compromise. Larger multicenter studies are warranted to further define the prognostic impact of PPHN in this population.

## Data Availability

The raw data supporting the conclusions of this article will be made available by the authors, without undue reservation.
